# Child-eating behaviour as predictor of anthropometric status of preschool children aged 2–4 years in Umuahia South LGA Abia State, Nigeria

**DOI:** 10.1186/s40795-024-00832-2

**Published:** 2024-03-07

**Authors:** Ijioma Okorie, Blessing K. Nwofia, Chinaza Ngonadi, Adaeze C. Okorie, Ngozi M. Nnam

**Affiliations:** 1 Department of Human Nutrition and Dietetics, Michael Okpara University of Agriculture Umudike, Umuahia, Abia State Nigeria; 2 Department of Dietetics, National Hospital Abuja, Abuja, Nigeria; 3 Department of Nutrition and Dietetics, University of Nigeria Nsukka, Nsukka, Enugu State Nigeria

**Keywords:** Preschool children, Anthropometric Status, Child eating behaviour, Gender

## Abstract

**Introduction and objective:**

Eating behaviour at the childhood level plays a vital role in the outcome of the nutritional status and the overall health of an individual. The study was focused on the association between anthropometric status and child eating behaviour.

**Methodology:**

A community-based cross-sectional survey purposively enrolled consenting participants from 256 households with preschool children aged 2–4 years. The parents/legal guardians were interviewed on the eating behaviour of their children using a validated semi-structured child-eating behaviour scale, and anthropometric measurement of the children were taken. WHO Anthro-software for child growth standards was used to categorize anthropometric status of the preschool children. Paired sample t-test was performed to compare child-eating behaviour by gender, while regression and correlation analysis was performed to determine the extent to which child-eating behaviour predicted anthropometric status at 5% level of significance.

**Results:**

Mean comparison of child eating behaviour by gender showed significant difference (*P* < 0.05) between male and female children in their eating behaviour with respect to enjoyment of food and satiety responsiveness. Some of the children were wasted (26.6%), stunted (20.7%) and underweight (16.4%). A significant association (*P* < 0.05) was observed between body mass index-for-age and food fussiness behaviour of the children. There was also a significant difference (*P* < 0.05) between weight-for-age and food fussiness behaviour of the children.

**Conclusion:**

The study showed that child eating behaviour may have contributed to the anthropometric status of the children, however, differences in their eating behaviours by gender was observed.

## Introduction

According to Okorie, Asumugha & Okorie [1], the prevention of obesity which is one of the nutrition-related disorders could be made possible through eating behaviour. The nutrition situation analysis in Nigeria showed that children under the age of five are most hit with malnutrition with Nigeria Demographic Health Survey (NDHS) [2] report showing that 37.0% of the children are stunted, 7.0% wasted, 22.0% underweight, while about 2.0% of under-five children are overweight and/or obese. Malnutrition affect under-five children and it is a global health challenge especially in developing countries of the world. More so, nutritional (anthropometric) status is an important global well-being, growth and development indicator [3]. Preschool age (2–4 years) is a period where growth rate is steady and also catchup could occur, once malnutrition is tackled and managed adequately [4]. Childhood is a time when food preferences and/or habits are formed. However, family influences play a critical role in food intake pattern, eating behaviour and obesity determination [1].

Eating behaviour is an action that is, governed by cognitive skills as a result of physiological, psychological and external factors with respect to food intake. Wardle et al. [5] opined that eating behaviour is classified into interest in food and lack of interest in food. A study observed that overweight children are interested in food, have greater response to food which makes them enjoy eating [6]. While children who are underweight are said to eat slowly thereby consumed small portion of food leading to lack of interest in food [6-8]. There are different kinds of eating behaviour in children which include satiety responsiveness (SR), slowness in eating (SE), food fussiness (FF), food responsiveness (FR), enjoyment of food (EF), desire to drink (DD), emotional under-eating (EUE) and emotional over-eating (EOE) [7]. It is known that eating behaviour are formed in the first years of life, representing behaviour traits that may change overtime, and that eating habits in adulthood are related to those learned in childhood [1, 9]., and as such could positively and/or negatively affect the nutritional status of an individual. Therefore, the study objective focused on child-eating behaviour as predictor of anthropometric status of preschool children aged 2–4 years in Umuahia South Local Government Area (LGA) of Abia State, Nigeria.

## Methodology

The study adopted cross-sectional and community-based design, through which associations between anthropometric status and child eating behaviour among preschool children 2–4 years were determined. Consent letter was obtained from the study participants’ mothers/caregivers and fathers of the preschool children, as well as the traditional ruler of the community.

### Study population

The study population was two hundred and ninety (290) preschool children aged 2–4 years living in communities in Umuahia South LGA of Abia State. The sample size for the study was estimated using Cochran’s formula elucidated by Araoye [10].

### Choice of respondents

The study aimed to investigate child eating behaviour among preschool children because this is the stage where eating behaviours are formed that would trickle into adulthood thereby influence their nutritional/anthropometric status. There is urgent need to curb malnutrition menace in all its forms, and hence the study, which promises to provide information that will guide partners, implementers, governments at all level, academia, civil society and private sectors to drive policy implementation to mitigate malnutrition amongst under-five children.

### Sample size determination

$$n\, = \,\frac{{{Z^2}P(100 - P)}}{{{e^2}}}$$

Margin of tolerable sampling error applied is 5% and 95% confidence level of the standard normal distribution curve which is Z = 1.96 will be used.

P = Prevalence of underweight among under 5 children in Nigeria which is 22% according to NDHS [2].

$$\begin{array}{*{20}{l}}{N{\mkern 1mu} = {\mkern 1mu} \frac{{{{1.96}^2}X22(100 - 7)}}{{{5^2}}}{\mkern 1mu} = {\mkern 1mu} \frac{{3.8416X22\left( {100 - 22} \right)}}{{25}} = {\mkern 1mu} \frac{{3.8416x1716}}{{25}}{\mkern 1mu} = {\mkern 1mu} 263.687{\mkern 1mu} }\\{\mkern 1mu} \end{array}$$

To make up for dropout during data collection, 10% of the sample size was computed and added to the sample size. 263.687 × 10% of 263.687 = 263.687 + 26.3687 290.056 = 290.

Therefore, the sample size was two hundred and ninety (290) preschool children.

### Sampling procedure

Prior to data collection, a non-probability purposive sampling technique was used to identify two hundred and ninety (290) households with preschool children aged 2–4 years across the five [5] communities in Umuahia South LGA. However, 256 households consented to participate in the study, and this gave 88.3% response rate (that is 256/290 × 100/1). One [1] child per household was recruited to be part of the study. In situations where a household had more than a child within the study age group, a simple random sampling by balloting without replacement was used. The ballot papers had only one “Yes” with several “No” and the children in such households were allowed to pick and the child that picked “Yes” was part of the study. However, this method was not applicable in households with only a child within the age bracket (2–4 years) for the study, and as such the child automatically becomes part of the study.

### Data collection

A pretested and validated semi-structured questionnaire was used to collect information on the date of birth of the preschool children, child eating behaviour and socioeconomic characteristics of their caregivers. For weight measurement, a Camry digital bathroom weighing scale was used to measure the children’s weight. The measurement was done with each child naked and barefooted, standing on the scale with head pointing straight. The weight was taken to the nearest 0.1 kg. The height measurement was carried out with calibrated height-meter (standio-meter), whereby each child was measured barefooted and the head, back, buttocks, calves and heels rested on the height rule for accurate reading of the height, while looking straight. Height measurement was recorded to the nearest 0.1 cm. Child-eating behaviour was assessed using the Child Eating Behaviour Questionnaire (CEBQ), which has eight items and contained thirty-five questions. The 8 CEBQ items include, food responsiveness (4 questions), emotional over-eating (4 questions), enjoyment of food (4 questions), desire to drink (3 questions), satiety responsiveness (5 questions), slowness in eating (4 questions), emotional undereating (4 questions) and food fussiness (7 questions).

### Data analysis

The World Health Organization (WHO) Anthro-Software for Child Growth Standards was used to categorize the anthropometric status of the preschool children into weight-for-age, height-for-age and weight-for-height z-scores ± 2 standard deviation (SD), while child eating behaviour was assessed using a 5-point Likert scale (Never = 1; Rarely = 2; Sometimes = 3; Often = 4; and Always = 5). The mean of the responses was computed and graded as poor and good eating behaviours. Mean of 1.00–2.99 = poor eating behaviour and while mean of 3.00 and above = good eating behaviour.

### Statistical analysis

Descriptive statistics– frequency and percentages was used to analyze the socioeconomic characteristics of their caregivers, and cross-tabulation was used to analyze the anthropometric status of the preschool children. Pair-sample t-test was used to determine differences in their eating behaviour by gender, while regression and correlation analysis was performed to determine the extent to which child-eating behaviour predicted anthropometric status, and also, regression analysis was performed to determine the extent to which socioeconomic status predicted eating behaviour at 5% level of significance. Data were presented in mean and standard deviation.

## Results

Table 1 presents the socioeconomic characteristics (education, occupation and income) of the parents and/or legal guardians. About 58.6% of the parents and/or legal guardians had tertiary education, while 27.3% and 5.1% had secondary and primary education respectively, and 9.0% of the caregivers had no formal education. The parents and/or legal guardians were traders (48.4%), skilled workers (19.5%), civil/public servants (14.1%), unemployed (8.6%) and housewives (9.4%). Amongst the parents and/or legal guardians, about 37.5% earned 30,000– 40,000 naira monthly, 18.8% of them earned 52,000 naira and above monthly, and while 30.1% and 13.7% of them earned less than 30,000 and 41,000– 51,000 naira, respectively, monthly.

**Table 1:** Socioeconomic characteristics of the parents and/or legal guardians of the preschool children

Parameters	Parents/legal guardians	
	Frequency	Percentage
**Educational level**		
No formal education	23	9.0
Primary education	13	5.1
Secondary education	70	27.3
Tertiary education	150	58.6
Total	256	100.0
**Occupation**		
Civil/public servant	36	14.1
Business/trading	124	48.4
Artisan/skill worker	50	19.5
Unemployed	22	8.6
House wife	24	9.4
Total	256	100.0
**Income**		
Less than 30,000	77	30.1
30,000-40,000	96	37.5
41,000-51,000	35	13.7
52,000 and above	48	18.8
Total	256	100.0

Table 2 presents the mean comparison of child eating behaviour by gender. Among all the child eating behaviour questionnaire items such as, food responsiveness, emotional overeating, enjoyment of food, desire to drink, satiety responsiveness, emotional undereating, slowness in eating and food fussiness, the result showed that differences existed in enjoyment of food and satiety responsiveness. There was a significant difference (*P* < 0.05) between male (3.68 ± 0.96) and female (3.41 ± 0.99) preschool children in enjoyment of food behaviour. However, a negative significant difference (*P* < 0.05) was observed between male (2.86 ± 0.74) and female (3.15 ± 0.79) preschool children in satiety responsiveness eating behaviour.

**Table 2:** Mean comparison of child eating behaviour by gender

Child eating behaviour	Male (*N* = 140)	Female (*N* = 116)	*p*-value
Enjoyment of food	3.68 ± 0.96	3.41 ± 0.99	0.029
Emotional over eating	2.03 ± 0.68	2.08 ± 0.64	0.568
Satiety responsiveness	2.86 ± 0.74	3.15 ± 0.79	-0.003
Slowness in eating	2.70 ± 0.64	2.84 ± 0.57	0.600
Desire to drink	3.23 ± 1.14	3.16 ± 1.23	0.610
Food fussiness	2.81 ± 0.55	2.87 ± 0.53	0.375
Emotional under-eating	3.16 ± 1.08	3.22 ± 1.15	0.691
Food responsiveness	2.79 ± 0.82	2.66 ± 0.84	0.219

Table 3 shows the anthropometric status of the preschool children. Weight-for-height result showed that 31.6% of the male preschool children had normal weight-for-height compared to 28.5% of their female counterparts. Also, 16.0% and 7.0% of the male children were wasted and severely wasted, respectively, while 10.5% and 6.2% of the female children were wasted and severely wasted, respectively. Result on height-for-age showed that 25.0% of the female children had a normal height-for-age compared to 23.8% of their male counterparts. There were also more stunted male children (12.1%) than stunted female children (8.6%). Similarly, 7.0% of male children were severely stunted compared to 5.9% of severely stunted female children. However, 11.7% of the male children were very tall for their age, while 5.9% of the female children were very tall for their age. There were 41.8% and 39.8%, respectively, of male and female children with normal weight-for-age, 11.3% (male) and 5.1% (female) of preschool children were underweight, and while 1.6% (male) and 0.4% (female) were severely underweight. The body mass index-for-age of the preschool children revealed that 10.5% (male) and 7.8% (female) were wasted, 6.2% (male) and 9.4% (female) were severely wasted, 5.9% (male) and 4.3% (female) were at risk of overweight, 3.1% (male) and 1.6% (female) were overweight, 1.6% (male) and 2.0% (female) were obese, and while 27.3% (male) and 20.3% (female) preschool children had normal body mass index-for-age in the study.

**Table 3:** Anthropometric status of the pre-school children

Parameters	Male F (%)	Female F (%)	Total F (%)
**Weight-for-height**			
Normal	81 (31.6)	73 (28.5)	154 (60.2)
Wasting	41 (16.0)	27 (10.5)	68 (26.6)
Severe wasting	18 (7.0)	16 (6.2)	34 (13.3)
Total	140 (54.7)	116 (45.3)	256 (100.0)
**Height-for-age**			
Normal height	61 (23.8)	64 (25.0)	125 (48.8)
Stunting	31 (12.1)	22 (8.6)	53 (20.7)
Severe stunting	18 (7.0)	15 (5.9)	33 (12.9)
Very tall for age	30 (11.7)	15 (5.9)	45 (17.9)
Total	140 (54.7)	116 (45.3)	256 (100.0)
**Weight-for-age**			
Normal weight	107 (41.8)	102 (39.8)	209 (81.6)
Underweight	29 (11.3)	13 (5.1)	42 (16.4)
Severe underweight	4 (1.6)	1 (0.4)	5 (2.0)
Total	140 (54.7)	116 (45.3)	256 (100.0)
**Body mass index-for-age**			
Normal BMI-for-age	70 (27.3)	52 (20.3)	122 (47.7)
Wasting	27 (10.5)	20 (7.8)	47 (18.4)
Severe wasting	16 (6.2)	24 (9.4)	40 (15.6)
Possible risk of overweight	15 (5.9)	11 (4.3)	26 (10.2)
Overweight	8 (3.1)	4 (1.6)	12 (4.7)
Obese	4 (1.6)	5 (2.0)	9 (3.5)
Total	140 (54.7)	116 (45.3)	256 (100.0)

F = frequency; % = percentage; BMI = body mass index

Table 4 shows the association between anthropometric status and child eating behaviour. Among the anthropometric status studied, body mass index-for-age and weight-for-age status had a relationship with preschool eating behaviour (food fussiness). There was a positive relationship (*P* < 0.05) between body mass index-for-age and food fussiness of the preschool children. The correlation between the body mass index-for-age and food fussiness was 0.201. There was also a positive significant relationship (*P* < 0.05) between weight-for-age status and food fussiness, with a correlation value of 0.183.

**Table 4:** Relationship between anthropometric status and child eating behaviour of the preschool children

(*N* = 256)	EOF	EOE	SR	SE	DD	FF	EUE	FR
**BAZ**								
r	-0.744	-0.084	0.064	0.019	-0.018	0.201	-0.011	-0.029
*p*-value	0.241	0.179	0.305	0.768	0.769	0.001**	0.864	0.642
**WAZ**								
r	-0.056	-0.044	-0.005	-0.012	0.063	0.183	-0.078	0.005
*p*-value	0.371	0.487	0.941	0.843	0.318	0.003**	0.211	0.933

EOF = enjoyment of food; EOE = emotional over eating; SR = satiety responsiveness; SE = slowness in eating; DD = desire to drink; FF = food fussiness; EUE = emotional undereating; FR = food responsiveness; BAZ = body mass index-for-age; WAZ = weight-for-age; *p* < 0.01

**Fig. 1:**
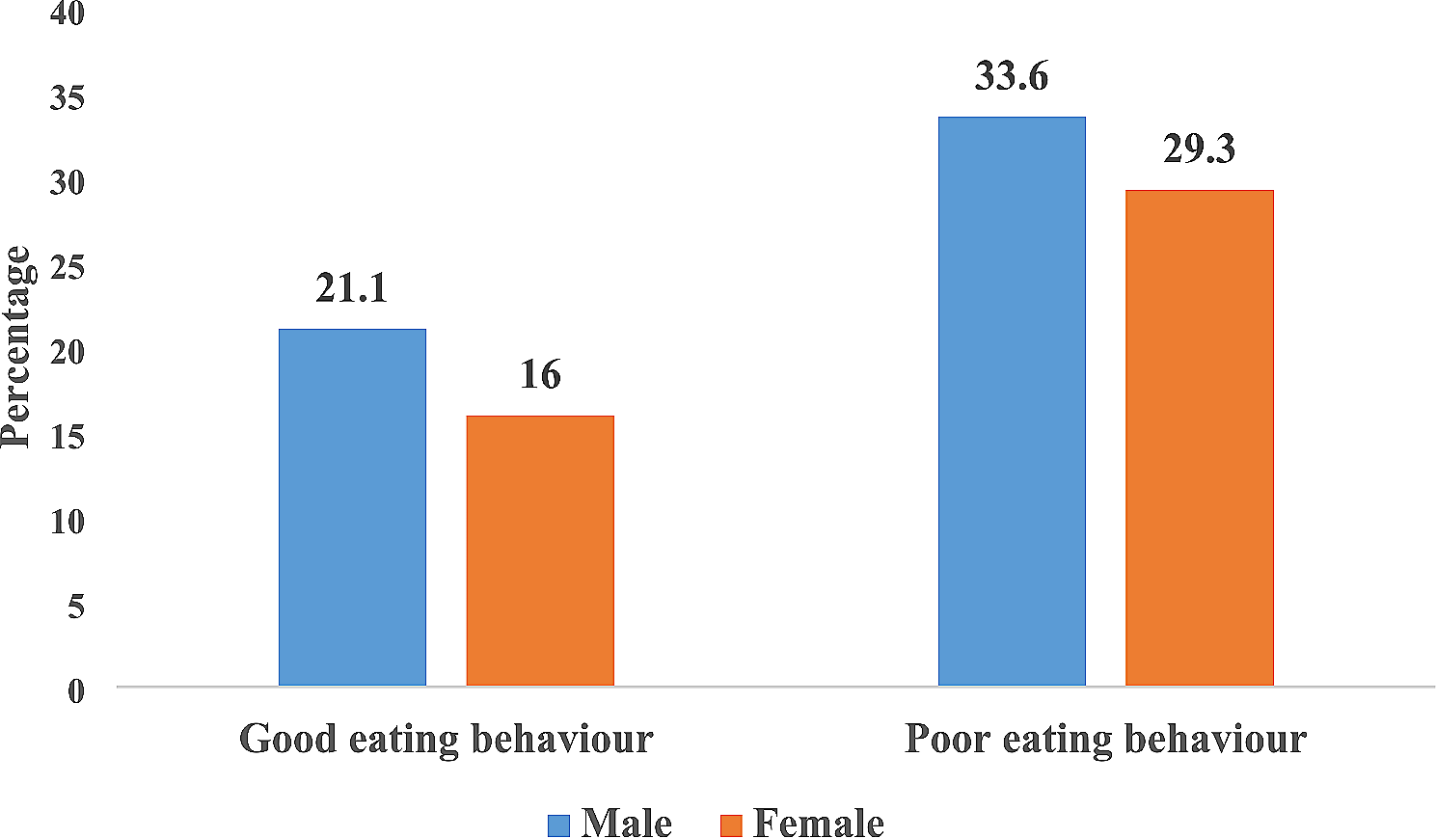
Child eating behaviour status of the preschool children by gender

Figure 1 shows the child eating behaviour status of the preschool children. The result showed that 21.1% (male) and 16.0% (female) of preschool children had good eating behaviour, while 33.6% (male) and 29.3% (female) of preschool children had poor eating behaviour. Table 5 shows that socioeconomic characteristics were a significant predictor of the child eating behaviour status of the preschool children in the study. The result however showed that among all the socioeconomic characteristics studied, having high income was the strongest predictor (*P* < 0.05) of child eating behaviour of the preschool children and contributed 2.2% of the variability in the child eating behaviour. Caregivers with high income status have preschool children with good child eating behaviour 14.4 higher than caregivers with low income status.

**Table 5:** Influence of socioeconomic characteristics on the child eating behaviour

Variables		Unstandardized Coefficients		R square	t	Sig.
		B	Std. Error			
	(Constant)	0.551	0.054			
**Child eating** **behaviour**	Low education	-0.109	0.090	0.022	-1.205	0.229
	Unemployed	-0.020	0.080		-0.248	0.804
	High income status	0.144	0.066		2.166	0.031

## Discussion

Majority of the mothers studied had tertiary education, and this could suggest that, in the study area the parents/legal guardians had access to education and this could in turn have impact on both family decision making and on the child’s eating behaviour. The level of education attainment in the present study was not similar to that reported by Opaluwah [11] who opined that women access to education is limited and/or constrained as a result household division of labour and workload. Furthermore, most of the parents and/or legal guardians were traders and also earned between 30,000 and 40,000 only per month. This could indicate that the parents and/or legal guardians engaged in a low paying trade despite attaining tertiary education level, and which may also be due to unavailability of white-collar jobs.

Enjoyment of food is an indicator of child eating behaviour and it is used to determine food approach behaviour among children, which could be a predisposing factor for obesity [12]. Thus, the study revealed that the male preschool children have a high degree of enjoyment of food than the female preschool children, which suggest gender-based individual differences with respect to eating behaviour such as enjoyment of food. The study is consistent with the study by Obidoa et al. [13] who opined that male school children tended to enjoy food more than female school children. However, the present study was not in line with the study by French et al. [12] who reported the relationship between food approach behaviour such as food enjoyment and obesity, because majority of the preschool children both male and female in the study were not obese, although they tended to enjoy food. Satiety responsiveness is an indication of food avoidance behaviour, and in the present study, the female preschool children were more satiety responsive to food than the male children. The study is not in agreement with the study by Obidoa et al. [13] who reported that boys were more satiety responsive to food than girls. The result further suggests that dysfunctional eating habits could be significantly linked with gender, which is in line with the study by Lipowska et al. [14].

Anthropometric indicators were used to detect malnutrition among preschool children in the selected communities. This is because malnutrition adversely affects weight and height in children. The study showed that there were some levels of wasting, stunting and underweight among the preschool children irrespective of gender in the study communities. This therefore, could be attributed to their failure to receive adequate nutrition/food intake over a short and long period of time, with poor availability of diversified diet, and disease. The study reported a high (26.6%) prevalence of wasting compared to that (9.0%) reported for Abia State by Nigeria Demographic Health Survey (NDHS) [2]. Although, it was an aggregated data (9.0%) for the State, the result of the study could suggest that low weight-for-height (wasting) is on the increase in the State presently. Stunting rate (20.7%) was lower compared to that (22.2%) reported for Abia State, while the underweight rate (16.4%) was higher than that (14.6%) at the State level by NDHS [2]. Thus, from the result of the study, it then, implies that malnutrition is spreading fast in some rural communities of Abia State. Food fussiness is an eating behaviour that is at peak during childhood, whereby children are unwilling to try and/or eat both known, unknown and/or new foods, and this can contribute to inadequate intake of varied nutrient-dense foods, and thus expose them to malnutrition, such as low body mass index-for-age and underweight. There was a strong relationship between food fussiness and the body weight of the preschool children. This could suggest that food fussiness may have contributed to the level of underweight and low body mass index for age (wasting) seen in the study.

Income plays a vital role in both the type of food purchased, eaten and also in eating habit and/or behaviour. It has earlier been reported that low-income households cut down on food purchase, thereby focusing on eating less quantity and quality food such as cheaper fast, junk or frozen food, when cost of living increases [15–17]. This will invariably affect the eating behaviour, health and nutritional status of individual member of the households. The study showed that caregivers’ high income contributed to good eating behaviour of the preschool children. Despite the high income of caregivers, the result suggests that preschool children eating behaviours were regulated by their caregivers with respect to both interest in food and lack of interest in food, in order to form a sustainable eating behaviour into adulthood, and subsequently achieve and/or maintain a good nutritional status [18].

## Conclusion

It was observed that child eating behaviour contributed to the anthropometric status of the preschool children in the study irrespective of gender. However, there were differences in eating behaviour by gender. Therefore, the result of the study validates that, eating behaviour is a veritable measure and/or tool needed to assess nutritional status of individuals with respect to socioeconomic status. Thus, there is need to scale up research on child eating behaviour with respect to diet quality, nutrient adequacy, and socioeconomic status of the parents and/or legal guardians.

## Data Availability

Data that support the findings of this study are not openly available due to some sensitivity and are available from the corresponding author upon reason request.
